# The multifaceted functions of DNA‐PKcs: implications for the therapy of human diseases

**DOI:** 10.1002/mco2.613

**Published:** 2024-06-19

**Authors:** Jinghong Wu, Liwei Song, Mingjun Lu, Qing Gao, Shaofa Xu, Ping‐Kun Zhou, Teng Ma

**Affiliations:** ^1^ Cancer Research Center Beijing Chest Hospital Capital Medical University/Beijing Tuberculosis and Thoracic Tumor Research Institute Beijing China; ^2^ Department of Thoracic Surgery Beijing Chest Hospital Capital Medical University, Beijing Tuberculosis and Thoracic Tumor Research Institute Beijing China; ^3^ Beijing Key Laboratory for Radiobiology Beijing Institute of Radiation Medicine Beijing China

**Keywords:** class switch recombination, DNA damage, DNA‐PKcs, innate immunity, V(D)J recombination

## Abstract

The DNA‐dependent protein kinase (DNA‐PK), catalytic subunit, also known as DNA‐PKcs, is complexed with the heterodimer Ku70/Ku80 to form DNA‐PK holoenzyme, which is well recognized as initiator in the nonhomologous end joining (NHEJ) repair after double strand break (DSB). During NHEJ, DNA‐PKcs is essential for both DNA end processing and end joining. Besides its classical function in DSB repair, DNA‐PKcs also shows multifaceted functions in various biological activities such as class switch recombination (CSR) and variable (V) diversity (D) joining (J) recombination in B/T lymphocytes development, innate immunity through cGAS–STING pathway, transcription, alternative splicing, and so on, which are dependent on its function in NHEJ or not. Moreover, DNA‐PKcs deficiency has been proven to be related with human diseases such as neurological pathogenesis, cancer, immunological disorder, and so on through different mechanisms. Therefore, it is imperative to summarize the latest findings about DNA‐PKcs and diseases for better targeting DNA‐PKcs, which have shown efficacy in cancer treatment in preclinical models. Here, we discuss the multifaceted roles of DNA‐PKcs in human diseases, meanwhile, we discuss the progresses of DNA‐PKcs inhibitors and their potential in clinical trials. The most updated review about DNA‐PKcs will hopefully provide insights and ideas to understand DNA‐PKcs associated diseases.

## Graphical abstract

DNA‐PKcs is involved in nervous system diseases, senescence, asthma, cardiovascular disease, cancers, chronic kidney diseases, and rheumatoid arthritis.

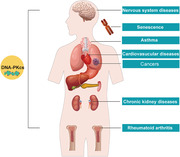

## 1 INTRODUCTION

In eukaryotic cells, various exogenous (ionizing radiation, chemical carcinogens, and ultraviolet radiation) and endogenous (e.g., DNA replication errors) insults are constantly threatening the genomic DNA^1^ and cause different forms of DNA damage including single or double DNA strand breaks, base damage, pyrimidine dimer, and so on. Among them, DNA double strand breaks (DSBs) is the most serious, which affects the genome stability and cell fate.^2^, ^3^ The eukaryotic DSBs repair pathways mainly include the homologous recombination (HR), nonhomologous end joining (NHEJ), and alternative end‐joining.^4^, ^5^, ^6^, ^7^

**FIGURE 1:**
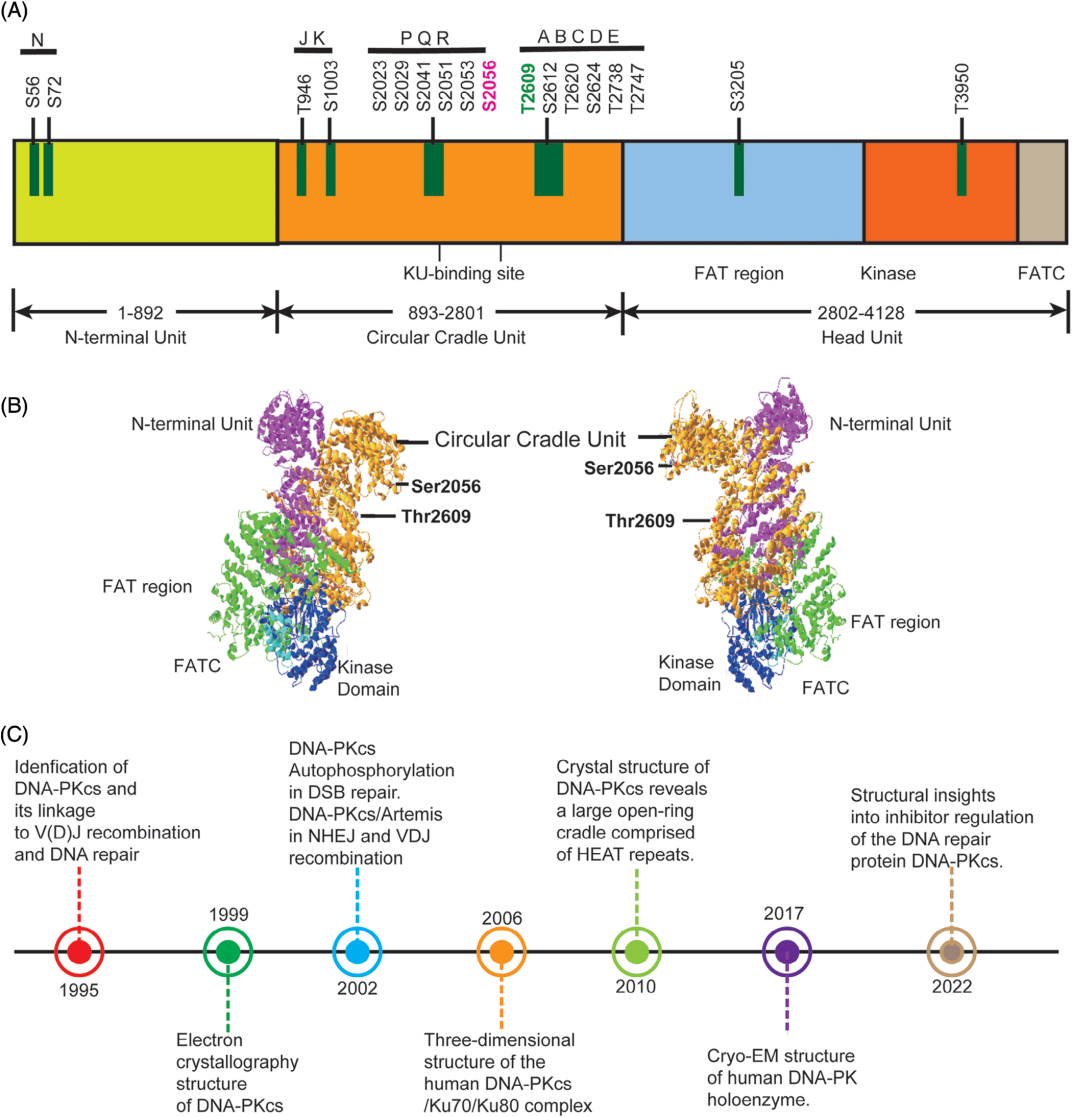
The functional domains and phosphorylation sites of DNA‐PKcs. (A) DNA‐PKcs contains a N‐terminal HEAT (Huntingtin, elongation, factor 3, protein phosphatase 2A, TOR1) repeats domain and a C‐terminal kinase domain surrounded by FAT (FRAP‐ATM‐TRRAP) and FATC (FATC‐terminal) domains. And the modification site of DNA‐PKcs. (B) The structure of DNA‐PKcs with functional domains and phosphorylation sites as depicted. (C) The history of key events in DNA‐PKcs discovery.

DNA‐PKcs is one of the most important players in the NHEJ pathway and also functions in multiple nodes of DNA damage response. DNA‐PKcs was first discovered about three decades ago. It is encoded by the *PRKDC* gene^8^ and is a part of SP1 transcription complexes.^9^ Initially, certain protein in the cell extracts was found to be phosphorylated after the addition of double‐stranded DNA (dsDNA), later DNA‐PKcs was identified when HSP90 or casein was used as phosphorylation substrate bait and DNA‐PKcs is ubiquitously expressed in cells^10^, ^11^ DNA‐PKcs is the member of the phosphoinositide 3‐kinase‐related protein kinases (PIKKs) family including Ataxia telangiectasia and Rad3 related (ATR) and Ataxia telangiectasia mutated (ATM). All these three PIKKs kinases contain a N‐terminal HEAT (Huntingtin, elongation, factor 3, protein phosphatase 2A, and yeast kinase TOR1) repeats domain, FAT (FRAP‐ATM‐TRRAP) domain, kinase domain, and FATC (FATC‐terminal) domain (Figure 1A).^12^, ^13^

**FIGURE 2:**
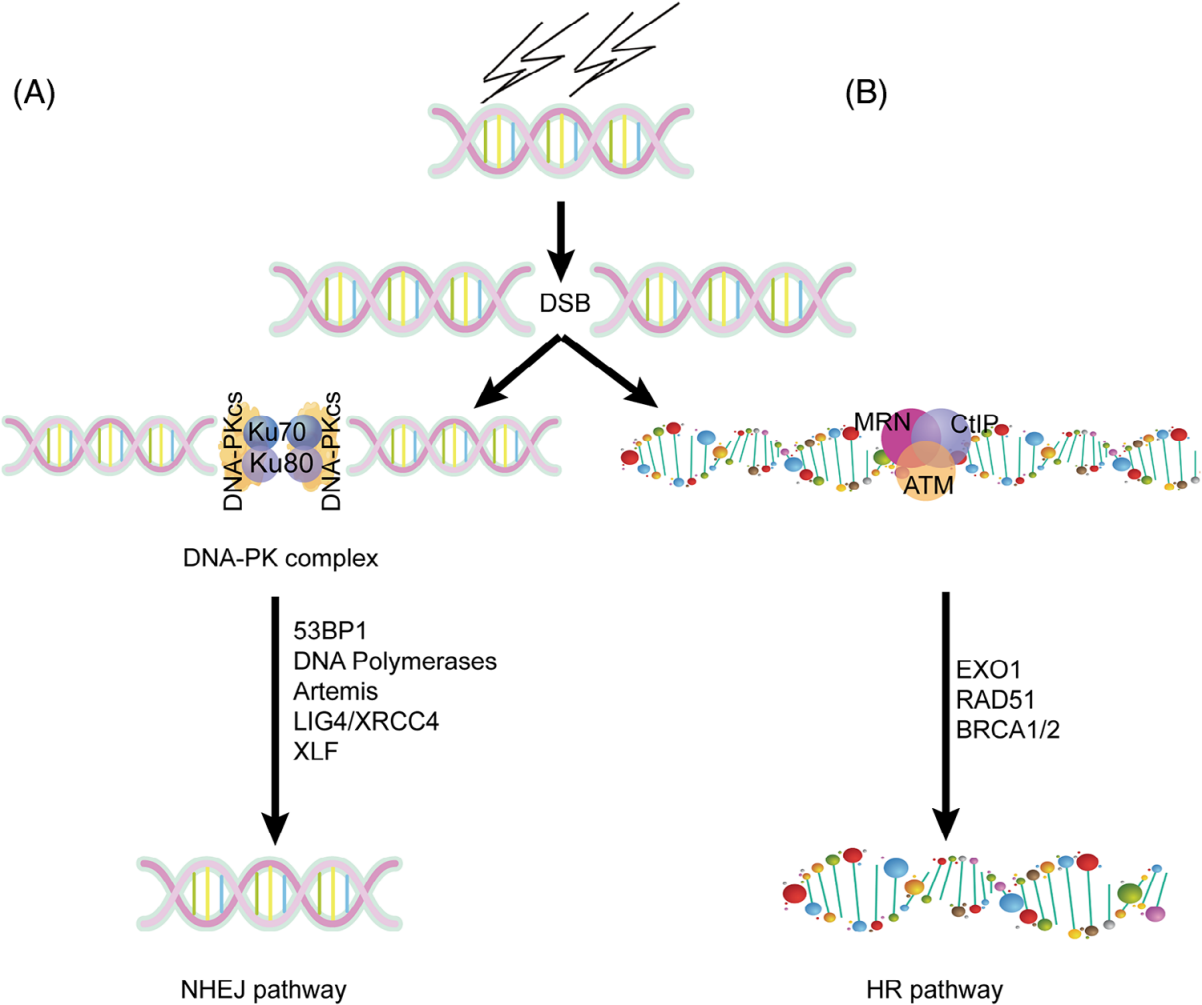
The DSB repair of NHEJ and HR. (A) The function of DNA‐PKcs in NHEJ pathway. In NHEJ, DNA‐PKcs and Ku70/Ku80 form a complex to bind to the site of DNA breaks and complete the repair of DNA breaks through the involvement of multiple factors such as 53BP1 and so on. This process usually occurs in G0/G1 phase. (B) In HR, MRN, ATM, and CtIP form a trimer, followed by the participation of EXO1, RAD51, and so on, to complete the repair of DNA. HR usually occurs in S/G2 phase. DSBs, double strand breaks; NHEJ, nonhomologous end joining; HR, homologous recombination; XRCC4, the X‐ray cross complementing protein 4; XLF, the XRCC4‐like factor; RAGs, recombination‐activation genes; BRCA1/2, breast cancer susceptibility genes1/2.

During DSB repair, the heterodimer of Ku70/Ku80 is recruited to the broken DNA ends rapidly, then DNA‐PKcs joins and DNA‐PK is activated. Then, other NHEJ factors including Artemis, XRCC4, DNA polymerase, and DNA ligase IV join the broken DNA ends. The DNA‐PK complex can stabilize the broken DNA ends and protect the broken DNA ends from resection by DNA exonucleases and subsequently accelerate broken end joining,^14^ as shown in Figure 2.

In addition to DSB repair, the immunoglobulin (Ig) diversity and B cell maturation process also require the canonical NHEJ (cNHEJ) pathway.^15^ Spontaneous mutations in DNA‐PKcs were found in patients showing severe defects in V(D)J recombination, CSR, and increased microhomology (MH) in the residual CSR junctions.^16^, ^17^ *PRKDC* gene mutations are also related to biomarkers that predict the efficacy of immunotherapy, such as microsatellite instability (MSI) and tumor mutation load.^18^, ^19^ Data from TCGA datasets indicate that samples with *PRKDC* mutation have remarkable increased infiltration of NK cells, CD8^+^ T cells, and increased expressions of immune checkpoint molecules, chemokines, and so on. Therefore, thorough understanding of DNA‐PKcs functions in immunity is critical to immune biology and has promising implications in clinic.

In this review, we will comprehensively summarize DNA‐PKcs structure and its function in DNA repair, immune system, immunodeficiency diseases, neurological disorders, and tumor development and progression. Furthermore, we will also discuss DNA‐PKcs as a candidate therapeutic target in human diseases and DNA‐PKcs inhibitors and their therapeutic potential. By highlighting DNA‐PKcs multifaceted roles in diseases, the review aims to provide insights into therapeutic targeting DNA‐PKcs and innovative treatments for cancer, immunotherapy, and other diseases. It also bridges recent advancements about DNA‐PKcs in both physiological and pathological contexts, paving the way for future directions.

## 2 OVERVIEW OF DNA‐PKcs STRUCTURE, FUNCTION IN DNA REPAIR

### 2.1 The structure of DNA‐PKcs

Efforts to resolve DNA‐PKcs structure has been making since its discovery in 1995 (Figure 1B,C). In 1999, DNA‐PKcs structure at 22 Å resolution was first determined by electron crystallography.^20^ In 2002, DNA‐PKcs/Artemis complex was found to participate in hair opening and overhang processing of NHEJ and V(D)J recombination.^21^ In the same year, DNA‐PKcs autophosphorylation was reported to function in NHEJ of DNA DSBs repair.^22^ Later in 2006, the three‐dimensional structure of human DNA‐PKcs/Ku70/Ku80 complex was revealed by single‐particle electron microscopy.^23^ The DNA‐PK holoenzyme dimer synaptic complexes were observed to contact DNA ends in proximity. In 2010, the DNA‐PKcs crystal structure at 6.6 Å resolution was reported.^24^ The HEAT repeats form a flexible cradle to promote DNA damage repair. In 2017, the structure of human DNA‐PK holoenzyme at 6.6 Å resolution was determined by cryo‐electron microscopy (cryo‐EM).^25^, ^26^ In 2022, the structure of human DNA‐PKcs in complex with four inhibitors (wortmannin, NU7441, AZD7648, and M3814) and adenosine‐5′‐(γ‐thio)‐triphosphate (ATPγS) were determined by cryo‐EM, which will greatly boost efforts in future drugs targeting DNA‐PKcs.^27^

### 2.2 The function of DNA‐PKcs in DNA repair

Structurally and biologically DNA‐PKcs is poised for NHEJ repair of DSB lesions.^28^, ^29^ DNA‐PK can form two distinct synaptic complexes: one promoting DNA end resection while the other promoting fill‐in end processing.^30^ DNA‐PKcs can be trans‐phosphorylated or auto‐phosphorylated on at least 40 sites located in different phosphorylation clusters such as JK, PQR, and ABCDE under the background of DNA repair.^31^, ^32^ Among them, PQR and ABCDE are the best characterized clusters, which is flanked by Ser2056 and Thr2609, respectively. Thr2609 was preferentially trans‐phosphorylated by ATR or ATM during replication stress or DSB repair.^33^, ^34^ DNA‐PKcs Thr2609 phosphorylation regulates Artemis access to DNA ends and is critical for Artemis‐mediated endonuclease activity and to promote subsequent HR.^35^, ^36^ Moreover, ATM‐mediated T4102 phosphorylation site was found in DNA‐PKcs protein,^37^ which promotes NHEJ. The Artemis protein harbors 10 DNA‐PKcs phosphorylation sites in its C‐terminal domain, which negatively regulate the Artemis endonucleolytic activities, and C‐terminal domain phosphorylation by DNA‐PKcs may rescue the inhibition (Figure 1A).^38^ When DNA‐PKcs activity is inhibited, ATM kinase activity can compensate for DNA‐PKcs autophosphorylation and promote resection. The Mre11–Rad50–Nbs1 (MRN) complex recruits EXO1 and further stimulates resection in the presence of Ku and DNA‐PKcs. It also suppresses end rejoining mediated by DNA ligase IV/XRCC4.^39^ The strict auto‐phosphorylation site Ser2056 is essential for NHEJ repair and response to DSB repair.^40^ In S phase of the cell cycle, BRCA1 blocks DNA‐PKcs autophosphorylation through interaction with DNA‐PKcs, thus allowing DNA end resection for HR.^41^ It is also found that upregulated activity of DNA‐PKcs can suppress HR repair in G1 phase of cells via increasing RBX1 protein expression. RBX1 promoted neddylation and activity of cullin1, and cullin1 is a key component of Skp1–Cullin1–Fbox ubiquitin 3 ligase, consequently mediating ubiquitination and degradation of EXO1.^42^ The interaction of TIP60 histone acetyltransferase and DNA‐PKcs prompts the autophosphorylation and activation of DNA‐PKcs.^43^

In addition to the automatic regulation of DNA‐PKcs, many other factors can regulate DNA‐PKcs activity, too. For example, epidermal growth factor (EGFR)^44^, ^45^ and the nonkinase regulator protein phosphatase 6 and protein phosphatase 1.^46^, ^47^ The deacetylase SIRT2 deacetylates DNA‐PKcs in response to DNA damage and subsequently promotes DNA‐PKcs interaction with Ku and the DNA‐PK activities.^48^ Small Cajal body‐specific RNA 2 (scaRNA2) weakens the interaction between DNA‐PKcs and Ku70/Ku80 through binding to DNA‐PKcs, thus inhibiting NHEJ while promoting HR.^11^

During replication stress, ATR phosphorylates DNA‐PKcs at the stalled replication forks and activates intra S‐phase checkpoint, through activation of Claspin transcription and stabilization of Chk1–Claspin complex.^49^ The apoptosis mediator p53‐induced protein with a death domain mediates DNA‐PKcs recruitment and promotes the ATR signaling pathway at the stalled replication forks.^50^

## 3 DNA‐PKCS AND HUMAN DISEASES

### 3.1 DNA‐PKcs in immune system and immunodeficiency diseases

**FIGURE 3:**
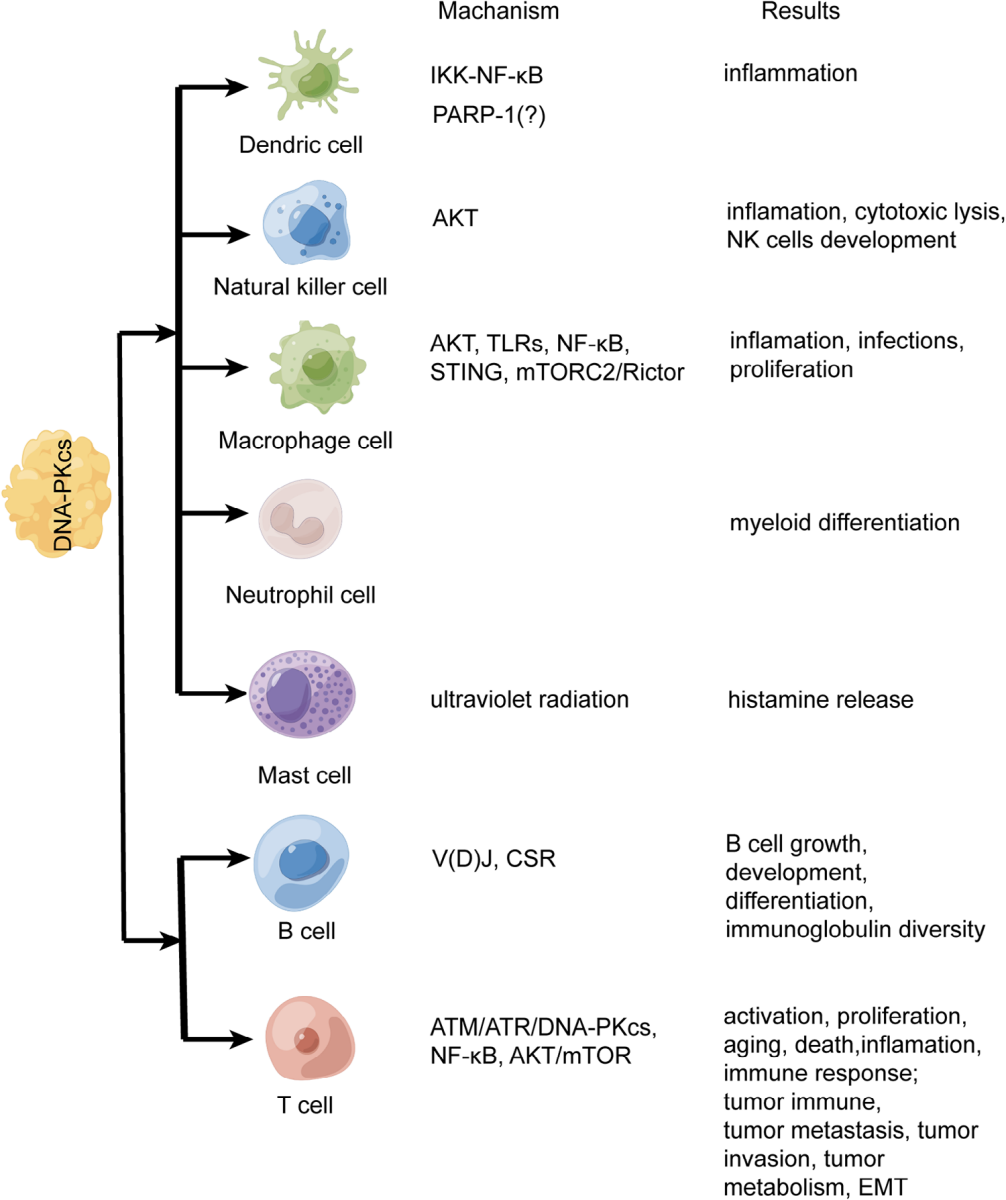
The regulation of DNA‐PKcs in immune cells. The regulation of DNA‐PKcs in immune cells. In DCs, DNA‐PKcs regulates inflammation via IKK‐NF‐κB, but whether the PARP‐1 is involved the regulation of DNA‐PKcs for DCs is uncertain. In NK, DNA‐PKcs regulates inflammation, cytotoxic lysis and development via AKT and other pathways. In macrophages, DNA‐PKcs regulates inflammation, infection and proliferation. In neutrophil cells, DNA‐PKcs regulates myeloid differentiation, but the mechanisms need exploration. In mast cells, DNA‐PKcs regulates histamine release under ultraviolet radiation. For B cells, DNA‐PKcs regulates cell growth, development, differentiation, and immunoglobulin diversity via V(D)J and CSR. For T cells, DNA‐PKcs regulates cell action, immune response, tumor, and so on via ATM/ATR/DNA‐PKcs and other pathways. DCs, dendric cells; NK cells, natural killer cells; V(D)J, variable (V) diversity (D) and joining (J); CSR, class switch recombination. Created with BioRender.com (https://www.biorender.com).

More and more evidences have supported that DNA‐PKcs regulates the immune system (Figure 3). Innate immunity is the first host defense against invading pathogens. Innate immunity has three basic functions: recognition, killing, and cleaning diverse pathogens. Innate immunity is mainly dependent on myeloid cells, which include mononuclear and poly‐morphonuclear phagocytes such as macrophage cells, dendric cells (DCs), natural killer cells (NK cells), and so on.^51^, ^52^ *PRKDC* mutation leads to SCID due to V(D)J recombination defect, and patients develop into autoimmune diseases because of overactivated innate immunity.^53^, ^54^, ^55^ DNA‐PK deficiency promoted antiviral innate immune responses in herpes simplex virus‐1 and vesicular stomatitis virus infection^56^; however, DNA‐PKcs suppresses Zika virus infection in human epithelial cells via acting downstream of IFNs transcription factors.^57^ A high concentration of anti‐DNA‐PKcs autoantibody can be detected in scleroderma, systemic lupus erythematosus patients, and so on.^58^, ^59^, ^60^ The autoimmune diseases related to DNA‐PKcs are listed in Table 1, so the role of DNA‐PKcs and drugs need to be further clarified in innate immunity.

**TABLE 1:** DNA‐PK with autoimmune disease.

Disease	Gender	DNA‐PK status	Clinical characteristics	References
Systemic lupus erythematosus	F and M	∖	Anti‐Ku, anti‐DNA‐PKcs, and antinuclear antibodies accumulations; XRCC7 6721G >T (rs7003908), XRCC6‐61C>G(rs2267437)	59
Polymyositis	∖	∖	Anti‐Ku antibody	61
Radiosensitive T‐B‐SCID	F	Mutation; normal DNA‐PK and DNA‐PKcs	Increased radiosensitivity and decreased DSB repair	62
SCID	M	Mutation; very low DNA‐PK and undetectable DNA‐PKcs	Growth failure; seizures; microcephaly; facial dysmorphism; bilateral sensorineural hearing loss; visual impairment; death.	63
SCID	F	*PRKDC* gene defect	Granuloma; diarrhea; skin lesion; papules; tachypneic; low CD4^+^ and CD19^+^ lymphocyte; low IgA and IgE	55
SCID	M	*PRKDC* gene mutation	Granuloma; asthma; acute arthritis; antinuclear antibodies; low IgG2, IgA, and IgG4; decreased T‐cell and B‐cell	60

Abbreviations: F, female; M, male.; SCID, severe combined immunodeficiency.

#### 3.1.1 DNA‐PKcs in DNA sensing pathways

DNA‐PKcs is thought to be a “sensor” for innate immunity and DSBs, which is crucial for eliminating pathogens. The SCID animals with DNA‐PKcs deficiency are highly susceptible to bacteria, viruses, and fungi infection.^64^ DNA‐PKcs can promote an antimicrobial innate immune response to foreign DNA involved in the secretion of IFN, cytokines, and chemokines.^56^, ^65^ DNA‐PKcs and Ku70/Ku80 are thought to comprise the dsDNA sensor for DSB repair through NHEJ.^66^ In DNA damage response, lysine‐516, lysine‐74, and lysine‐539 of Ku70 can be methylated by the methyltransferase SMYD2, facilitating the recruitment of Ku70/Ku80/DNA‐PKcs complex. The SMYD2 inhibitor treatment results in accumulation of cytosolic DNA, leading to activation of cGAS‐STING pathway.^67^ KU‐mediated DNA sensing facilitated DNA‐PKcs recruits in aging‐related autoimmunity.^68^ Without MRN, DSB repair can activate ATM. DNA‐PKcs efficiently substitutes for ATM in promoting local chromatin responses.^69^ DNA‐PK, neither ATM nor ATR, negatively interfere with HIV‐1 integration and lentivirus integration.^70^, ^71^ DNA‐PK suppresses enzymatic activity and phosphorylates cGAS; however, DNA‐PKcs deficiency increases cGAS and upregulates immune responses that restricts viral replication.^56^ Whether DNA damage sensors PARP‐1, DNA‐PK, ATM, and ATR modulate retroviral integration site selection during HIV infection and what other cellular proteins serve as integrase cofactors remain further exploration. Cytosolic accumulation DNA and subsequent DNA sensing pathways activation can lead to the development of autoimmune diseases because these patients presented with autoantibodies of DNA‐PK and RNA‐Pol III disorders the link between autoimmune diseases and DNA sensing.^72^

#### 3.1.2 DNA‐PKcs in innate immunity

##### 3.1.2.1 DNA‐PKcs in DCs

DCs are a class of immune cells that originate from hematopoietic stem cells and as a link between innate immunity and adaptive immunity. Their main function is antigen presenting.^73^ Ma et al.^74^ found that DNA‐PKcs was involved in the upregulation of JNK and IKK‐NF‐kB in oligodeoxynucleotides containing CpG motif signaling pathway. Depletion of DNA‐PKcs impaired the CpG‐A‐induced IFN‐α response in DCs.^75^ We can summarize that DNA‐PKcs is a significant mediator between TNF and Toll‐like receptors (TLRs). At the same time, Poly (ADP‐ribose) polymerase‐1 (PARP‐1) regulates the innate immunity thorough regulating gene transcription, modulating the ability of DCs.^76^ PARP1 can be phosphorylated by DNA‐PK, which leads to PARP1 cytoplasmic translocation^77^; however, related research confirming that DNA‐PKcs regulates DCs function via PARP‐1 is lacking. Intracellular reactive oxygen species (ROS) induced DNA‐PKcs phosphorylation in DCs, and pharmacological inhibition or specific abolishment of DNA‐PKcs in DCs attenuated anaphylaxis, indicating that DNA‐PKcs is a promising target in the treatment of allergic asthma.^78^

##### 3.1.2.2 DNA‐PKcs in NK cells

NK cells are CD34^+^ hematopoietic progenitor cells‐derived large granular lymphocytes. They secrete cytokines and chemokines that regulate the host immune response, kill infected cells, or abnormal cells via death receptor pathway or perforin/granzyme.^79^, ^80^ As a regulator, DNA‐PKcs could stimulate IFN‐γ production and participate in cytotoxic lysis by NK cells.^81^, ^82^ Knockout of *PRKDC* impaired lymphoid development and reduced the production of NK cells.^83^ DNA‐PKcs also participated in the biological responses of CD158d, which is defined as a killer cell specific Ig‐like receptor (KIR)2DL4(KIR2DL4). CD158d is an activation receptor for HLA‐G, which promotes inflammation and angiogenesis in NK cells, and CD158d interacted with DNA‐PKcs, thereby promoting the recruits of Akt to endosomes and phosphorylation of Akt via DNA‐PKcs‐dependent pathway.^84^, ^85^ Further study showed that CD158d agonists stimulate a DNA damage response and continuous activation of CD158d‐induced morphological changes of NK cells, as well as changes in shape and size, and accelerated cellular senescence; however, the interaction domains and specific sites between DNA‐PKcs and CD158d are unknown.^86^

##### 3.1.2.3 DNA‐PKcs in macrophage cells

As myeloid immune cells, macrophages are found throughout the body, where they ingest and degrade foreign materials, debris, aberrant cells, and participate in the inflammatory processes and TME functions.^87^ CpG motifs (CpG) is a stimulator of innate immune response. DNA‐PKcs directly phosphorylated and activated Akt upon CpG‐DNA exposure in macrophages.^88^ CpG collaborated with DNA‐PKcs to promote the production of IL‐10. DNA‐PKcs inhibitor, wortmannin, completely suppressed CpG‐induced IL‐10 secretion in macrophages.^89^ Meanwhile, DNA‐PK binds to the autoimmune regulator (AIRE), which is phosphorylated by DNA‐PKcs at residues Thr68 and Ser156. DNA‐PKcs and AIRE cooperate to upregulate the expression of TLRs in RAW264.7 cells.^90^ DNA‐PKcs regulated the genetic program, induced activation of inflammasome and the secretion of IL‐1β and IL‐18 in the infection of Listeria monocytogenes, whereas DNA‐PKcs‐deficient murine macrophages secrete low levels of IL‐18.^81^

Ku70/Ku80 heterodimer is pivotal for the function of DNA‐PKcs.^33^ It binds to DNA break ends, initiates NHEJ repair, recruits, and activates DNA‐PKcs.^91^ Ku70/Ku80 coimmunoprecipitated with p65‐p52 NF‐κB complex in macrophages and induced NF‐κB activation.^92^ Ku70–STING pathway is necessary to mediate DNA‐induced IFN‐λ1 production by macrophages and THP‐1 cells.^93^ Ku70 functions as a DNA sensor to interact with ssDNA90 and triggers innate immune responses to inhibit the human T lymphotropic virus type 1 protein expression in human monocyte‐derived macrophages, PMA‐stimulated THP1 cells, and primary human monocytes^94^; however, whether DNA‐PKcs is involved in these processes is unclear. According to recent study, apoptotic cell can be cleared by macrophages and the nucleotides derived from the hydrolytic cell DNA by phagolysosomal DNase2a can upregulate Myc to promote macrophage proliferation via DNA‐PKcs–mTORC2/Rictor pathway.^95^ Therefore, we can conclude that DNA‐PKcs regulate the immune function and proliferation of macrophages.

##### 3.1.2.4 DNA‐PKcs in neutrophil cells

Neutrophil cells are a type of myeloid leukocyte, which is the first responders. Neutrophils are the initial host defense against pathogens including cancer, bacteria, fungi, and protozoa.^96^, ^97^, ^98^ The expression of DNA‐PKcs in neutrophil is lower than lymphocytes, but DNA‐PKcs regulates myeloid differentiation.^91^, ^99^ DNA‐PKcs effects hematopoiesis and NHEJ play a critical role for somatic cell reprogramming and maintenance of genomic stability in induced pluripotent stem cells,^91^, ^100^ but whether DNA‐PKcs participates in the differentiation, development, and maturation of neutrophil cells remains to be further clarified. On the other hand, many studies have confirmed that neutrophils are plastic and polarizable in the tumor microenvironment^101^ and involved in tumor metastasis.^102^ There is no evidence whether DNA‐PKcs is involved in the regulation of neutrophils in tumors.

##### 3.1.2.5 DNA‐PKcs in mast cells

Mast cells (MCs) are tissue resident and is important in host defense, homeostatic response, and initiate type I allergic reactions.^103^ MCs can release histamine and various cytokines to regulate immune‐related responses such as bacterial infections, autoimmune diseases, inflammatory diseases, cancers, and allograft rejections.^104^ As versatile effector cells, MCs regulate tissue remodeling and repair.^105^, ^106^ Ultraviolet radiation (UVR) can activate MCs and induce histamine release. It is known that DNA‐PKcs is an important mediator to recover DSB repair under UVR, so it is interesting whether DNA‐PKcs regulate MCs function. In inflammatory responses, IL‐10, IL‐6, IL‐12, and TNF are involved in MCs development,^107^, ^108^ and DNA‐PKcs is involved in production of these inflammatory cytokines in macrophages and DCs.^74^, ^89^ It is necessary to clarify whether DNA‐PKcs regulates the production of inflammatory cytokines in MCs. In cancers, MCs can be attracted by tumor‐associated macrophages in the TME^109^ and upregulate tumor angiogenesis and lymphangiogenesis by releasing classical and nonclassical angiogenic factors.^110^ As a protumor protein kinase, DNA‐PKcs promotes the activation of VEGF transcription to upregulate migration, invasion, and tube formation in various cancers.^111^, ^112^ The crosstalk between DNA‐PKcs and MCs is still unsolved mystery. The function and regulation of DNA‐PKcs in DCs, NK cells, macrophages, neutrophil cells, MCs, T cells, and B cells are shown in Figure 3.

#### 3.1.3 DNA‐PKcs in adaptive immunity

**FIGURE 4:**
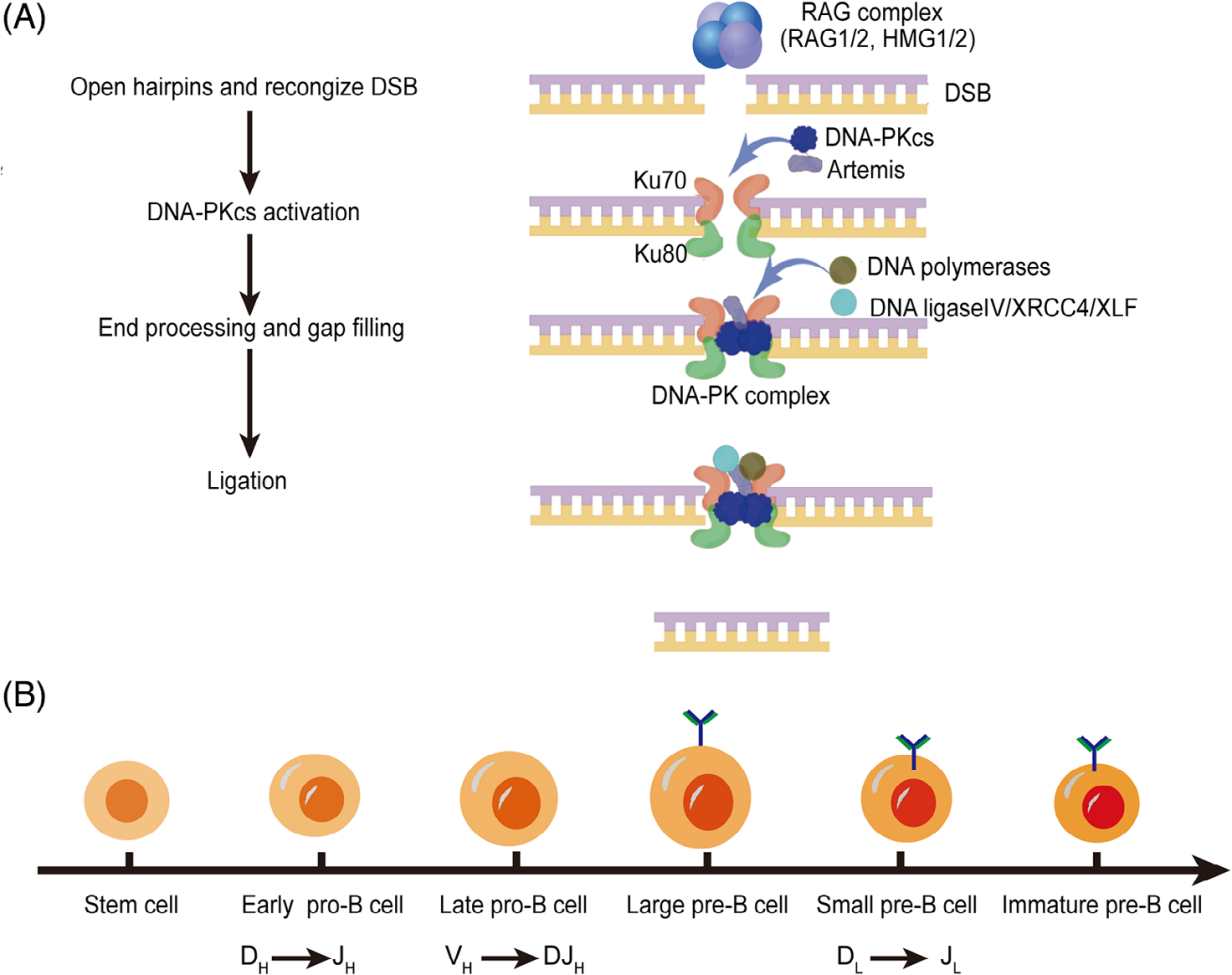
DNA‐PKcs in V(D)J recombination. (A) NHEJ pathway in V(D)J. the process as four steps: (1) RAG‐1/2 proteins combine HMG1 or HMG2 to form RAG complex, then the RAG complex open free hairpins. (2) The KU complex recognizes the DSBs, recruits the DNA‐PKcs, and together tether the break DNA ends. (3) Nucleases and polymerases participate in the processing of the DNA ends123. (4) The ligation of the processed DNA ends, conducted by XLF, DNA ligase IV, and XRCC4 in NHEJ. (B) The influence of DNA‐PKcs on B cells development and differentiation. DSBs, double strand breaks; RAGs, recombination‐activation genes; XRCC4, the X‐ray cross complementing protein 4; XLF, the XRCC4‐like factor. Created with BioRender.com (https://www.biorender.com).

Adaptive immunity has evolved to respond in a broader and more precise way for both self‐antigens and non‐self‐antigens. The main cells include T lymphocytes and B lymphocytes, which get mature in the thymus and arise in the bone marrow, respectively.^113^, ^114^ DNA‐PKcs has been proved to play a pivotal role for immune system, which mediates B and T cell maturation in all vertebrates (Figure 4). DNA‐PKcs coordinates with PARP‐1 to link p53 and IFN‐γ under the action of Trp‐tRNA synthetase in zebrafish.^115^, ^116^, ^117^ DNA‐PKcs maintains more genomic stability, longer lifespan, and mediates DNA repair in naive T cells than memory T cells from young subjects but not from elderly subjects.^118^ In aging‐related autoimmunity disease, DNA‐PKcs participates in activation of T cells, which aggravates the pathology of experimental autoimmune encephalomyelitis in aged mice.^117^ Enrichment of tumor immunogenicity microenvironments was found in solid tumors harboring *PRKDC* mutations, such as NK cells, CD8^+^ T cells, and chemokines. Importantly, enhanced the efficacy of ICI was observed after DNA‐PKcs inhibitor treatment and *PRKDC* mutations were associated with better survival in patients after ICI treatment, highlighting DNA‐PKcs as a potential biomarker for immunotherapy.^19^, ^119^ Multiple DNA‐PKcs inhibitors have been investigated for tumor immunotherapy.^120^ Studies involved these inhibitors are mostly in cancers as shown in Table 2, but little is known about DNA‐PKcs inhibitors in aging, autoimmune diseases, and infectious diseases, and will be the focus of future research.

**TABLE 2:** DNA‐PKcs inhibitors and diseases.

Inhibitor	Disease	Con.	Mechanism	Result	References
Wortmannin	Bladder cancer	10 µM	DNA‐PK↓, p‐P53↓, apoptosis↑, G2/M arrest	DSB repair↓	239
NU7026	NSCLC	10 µM	ATM↑, ATR↑, G2/M arrest, DNA‐PKcs↓, apoptosis↑	Radiosensitivity↑	240
	Glioblastoma and medulloblastoma	10 µM	p‐DNA‐PKcs (Ser2056)↓, proliferation↓	Telomere dysfunction, DSB repair↓	241
	Osteosarcoma	10 µM	apoptosis↑, Beclin‐1↓	Xenograft tumor growth↓, salinomycin's sensitivity↑	242
NU7441	Breast cancer and cervical cancer	1 µM	G2/M arrest, apoptosis↑, colon formation↓	Tumor growth↓, radiosensitivity↑	243
	B‐cell chronic lymphocytic leukemia	1 µmol/L	p‐DNA‐PKcs (Ser2056)↓	Mitoxantrone's sensitivity↑	244
	Allergic asthma	0.5 µM	AKT↓, mite antigens↓, p‐DNA‐PKcs (Ser2056)↓	Th2 inflammation↓	78
NU5455	human orthotopic lung tumors	30 mg/kg	∖	Doxorubicin's sensitivity↑, xenograft tumor volume↓	245
VX‐984	Glioblastoma	250–500 nM	Colon formation↓, p‐DNA‐PKcs (Ser2056)↓	Radiosensitivity↑	217
AZD7648	NSCLC	91 nM	cell growth↓, apoptosis↑, p‐DNA‐PKcs (Ser2056)↓, γH2AX↓	Olaparib efficacy↑, radiosensitivity↑	224

Abbreviations: DSBs, double strand breaks; NSCLC, non‐small cell lung cancer.

Igs are one of the major components of adaptive humoral immunity. The Ig diversification derives from RAG1/2‐mediated V(D)J recombination and AID‐mediated CSR, and somatic hypermutation (SHM).^121^, ^122^ V(D)J recombination is a process to generate functional B‐cell (Ig) and T‐cell receptors^123^, ^124^ and naive B lymphocytes can transform into different isotypes under the antigen stimulation through CSR.^16^ Next, we will discuss the role of DNA‐PKcs in V(D)J and CSR.

##### 3.1.3.1 DNA‐PKcs in Ig V(D)J recombination

The ability of mammalian immune system to identify numerous antigens relies on the antigen‐binding regions encoded by highly diverse antigen receptor genes on the surface of lymphocytes. Genes encoding antigen‐binding regions exist in a special form of gene clusters in the whole genome. Starting from the 5′ end of DNA, a series of variable, diversity, and joining gene segments are arranged. The length is about 2.5 Kbp to 3Mbp. The site‐specific V(D)J recombination process rearranges individual V, D, and J gene segments that are ready for recombination to form the gene encoding the antigen‐binding region. The mechanism of V(D)J recombination not only plays a key role in lymphocyte development, but also plays an important role in genomic instability and the occurrence of cancers.^125^, ^126^, ^127^ V(D)J recombination occurs in an inversional manner for almost half of the Ig light chain genes, and to keep chromosomal integrity both signal and coding joint must be formed. Signal ends are joined by Ku, XRCC4, and DNA ligase IV to form a signal joint. Then Artemis and DNA‐PKcs cooperate to open the hairpin, followed by end processing mediated by nucleolytic enzymes and polymerases. Finally, the two coding ends will be joined together to form a coding joint.^21^

Both CSR and V(D)J recombination depend on the NHEJ pathway. The V(D)J recombination is the important mechanism to produce Ig diversity because of error prone nature to match different pathophysiological environments.^15^ Ku70, Ku80, Artemis, DNA‐PKcs, DNA ligase IV, XLF, and XRCC4 are main elements of NHEJ pathway during V(D)J recombination,^128^, ^129^ among them, DNA‐PKcs is required for open coding ends and blunt signal ends.^31^, ^130^, ^131^ DNA‐PKcs inactivated SCID mice are unable to undergo the V(D)J recombination of T‐cell receptor genes and Ig.^132^ DNA‐PKcs mutation decreases the levels of DNA‐PKcs protein and affects the coding joint formation more severely than signal joining formation in murine.^133^, ^134^, ^135^, ^136^ DNA‐PKcs inhibitor M3814 (also known as Nedisertib) treatment leads to a remarkable reduction in coding joint formation compared to signal joint formation.^137^ However, other studies demonstrated that signal joining ineffectiveness in cells with DNA‐PKcs dysfunction is as severe as coding joint formation.^138^, ^139^, ^140^, ^141^ The difference among these studies may result from different cell types, different DNA‐PKcs mutations status and signal joining at endogenous antigen receptor versus extrachromosomal recombination substrates. Besides, Ku70 and Ku80 are required for repair of both signal and coding ends.^142^ The Ku70 and Ku80 heterodimer binds to exposed DNA ends and recruits DNA‐PKcs, which undergoes autophosphorylation and phosphorylates Artemis. Artemis gains various nuclease activities including hairpin opening activity and endonuclease activity for end processing.^15^, ^17^, ^120^ It plays roles during the V(D)J recombination and is indispensable for nucleotide loss from signal ends during the repair process.^143^ The hairpin structure is not resolved in Artemis^−/−^ mice and coding joint formation is severely inhibited.^144^ XRCC4 that can be phosphorylated by DNA‐PKcs interacts with DNA ligase IV, exerts biological activity in ligating both signal and coding ends.^129^, ^145^ Loss of XRCC4 leads to an increased dependence on MH‐mediated DNA repair during V(D)J recombination and increased sensitivity to DNA damaging agents,^142^ and abolishing XRCC4′s affinity for XLF results in a decrease of coding joint formation.^146^ Therefore, these elements of NHEJ pathway during V(D)J recombination are necessary and we can summarize this process as four steps: (1) RAG1/2 proteins combine HMG1 or HMG2 to form RAG complex, then the RAG complex open free hairpins. (2) The KU complex recognizes the DSBs, recruits the DNA‐PKcs, and together tether the DNA break ends. (3) Nucleases and polymerases participate in the processing of the DNA ends.^147^ (4) The ligation of the processed DNA ends, performed by XLF, DNA ligase IV, and XRCC4 in NHEJ,^148^ as shown in Figure 4.

##### 3.1.3.2 DNA‐PKcs in CSR

CSR is defined that Ig class switching from IgM (µ) to IgA (α), IgG (γ), or IgE (ε) happens as an intrachromosomal DNA deletion.^149^, ^150^ CSR event involves the generation of DSB repair and joining between the donor (µ) and acceptor (γ, ε, or α) S regions to generate the new arrangement.^151^ cNHEJ is the main DSB end‐joining pathway during CSR.^121^ The cNHEJ pathway is utilized by naive B cells to generate different isotypes of antibodies by joining two DNA double‐strand breaks at different switching regions via CSR.^17^ Besides the core cNHEJ factors, Artemis and DNA‐PKcs are also important for joining AID‐initiated DSB repair during CSR.^152^ Moreover, DNA‐PKcs could function independently of Artemis during CSR.^153^ Severe defects in both V(D)J recombination and CSR and increased MH in the residual CSR junctions were found in patients with spontaneous mutations in DNA‐PKcs.^62^, ^154^ Overall, DNA‐PKcs is indispensable in the process of CSR and Ig V(D)J recombination during B cell development. It should be noted that we need to further investigate the mechanism by which DNA‐PKcs involves in Ig diversity especially in CSR. DNA‐PKcs is essential for normal levels of p53 phosphorylation, apoptosis, and chromosomal stability in B and T cells.^155^ DNA‐PKcs^−/−^ mature B cell with assembled Ig heavy (IgH) and light chains (IgL) undergo a severely impaired CSR to all IgH isotypes except IgG1.^152^, ^156^, ^157^, ^158^ The kinase‐dead DNA‐PKcs (DNA‐PKcs^KD/KD^) show reduced CSR to all isotypes, including IgG1,^16^ indicating that a significant and direct role for DNA‐PKcs in CSR. However, B cells derived from SCID mice with inactivated DNA‐PKcs domain were found to switch from IgM to IgG or IgA as same as B cells from wild‐type, which may result from the different background of the transgenic mice,^156^, ^159^, ^160^ and DNA‐PKcs protein with kinase‐deficiency in B cells could play an important role in CSR, and the role of DNA‐PKcs in CSR should be separated from its role in NHEJ, which needs a functional kinase domain.

There are some modification sites of DNA‐PKcs such as S2056, T2609, T2647, K4007, and so on; among them, T2609 alters the repair pathway choice during CSR, S2056 promotes end ligation in cNHEJ, and T3950 knocks down DNA‐PKcs activity. Whether S2056 and T3950 are paradoxical during CSR remains elusive.^17^, ^120^, ^161^ More modification sites of DNA‐PKcs need to be verified.

To sum up, DNA‐PKcs has a direct and effective, but dispensable role in CSR. The reasons are: (1) Ku70 and Ku80 and ARTEMIS are important for CSR, too.^162^ (2) Without cNHEJ factors, CSR can be regulated by the alternative end‐joining (Alt‐EJ) pathways, which preferentially use MH.^163^, ^164^ Thus, although most of the available evidence points to the role of DNA‐PKcs for NHEJ in CSR, elucidating the role of XRCC4 or DNA ligase IV will provide the clearest evidence.

##### 3.1.3.3 DNA‐PKcs in T cells

In mature lymphocytes, DNA‐PKcs is robustly expressed and acts as key linker between T cells activation and fate.^118^, ^165^, ^166^ For T cells development, DNA‐PKcs participates in mitosis. It has been reported that PLK1 functions as a central regulator of mitosis from the mitotic entry to exit. It interacts with other mitosis‐related proteins within specific subcellular structures.^167^ Zhou and coworkers^168^ demonstrated that PLK1interacts with phosphorylated DNA‐PKcs throughout the mitotic phase and inactivation of DNA‐PKcs resulted in the dysregulation of mitotic progression, especially abnormal chromosome segregation and the cytokinesis failure. DNA‐PKcs is also critical for the stabilization of centrosome and spindle structure, and for the regulation of mitotic progression under DNA damage.^169^ DNA‐PKcs regulates the CD4^+^ T cells differentiation into Th1 and Th2 cells by Gata3 and Tbet in asthma, whether the similar mechanisms exist in other diseases remains to be explored.^166^ As we can see, DNA‐PKcs functions in cell cycle, mitotic progression, and the normal structure of chromosome; however, whether DNA‐PKcs directly participates in mitotic progression of T cells development or through other molecules such as BRCA1/2 and XRCC4 should be further clarified. We should focus on how DNA‐PKcs regulates T cells differentiation and proliferation. For T cells activation, resting T cells are hypersensitive to DNA damage through the activation of ATM/ATR/DNA‐PKcs pathway.^170^ DNA‐PKcs phosphorylates early growth response protein 1 (Egr1) at serine 301, which activates T cell and dictates T cell response by stimulating costimulatory molecules and cytokines such as interleukin (IL)‐2, IL‐6, IFNγ, and NF‐κB.^115^ Loss of DNA‐PKcs activity remarkably reduces the expression of IL‐6 and NF‐κB^171^ and DNA‐PKcs inhibition blocks calcineurin activity to prevent NFAT translocation to the nucleus and expression of cytokine IL‐2.^165^ Mechanistically, DNA‐PKcs‐mediated NF‐κB response to TNF‐α or IL‐1β might depend on phosphorylation of the p50 subunit at serine residue 20 by DNA‐PKcs, which appears to significantly affect the interaction between the p50 and p65 subunits of NF‐κB.^172^ DNA‐PKcs is necessary for mitochondrial function and homeostasis. Fan and coworkers^173^ reported that knockout DNA‐PKcs can improve mitochondrial metabolism to protect myocardium, liver, and kidney from sepsis. Further studies showed that DNA‐PKcs is involved in oxidative stress, it can interact with mitochondria proteins ANT2 and VDAC2 to form DNA‐PKcs/ANT2/VDAC2 complex to support exchange of ADP and ATP across mitochondrial membranes and maintain mitochondria membrane potential, and during this process, Thr2609 cluster of DNA‐PKcs is phosphorylated by ATM kinase.^34^ So we can conclude that DNA‐PKcs is vital for oxidation of cells and speculate that it takes part in oxidative reactions and energy metabolism and it can be a potential target for the treatment of metabolic disorders.

#### 3.1.4 DNA‐PKcs in immune‐related diseases

**FIGURE 5:**
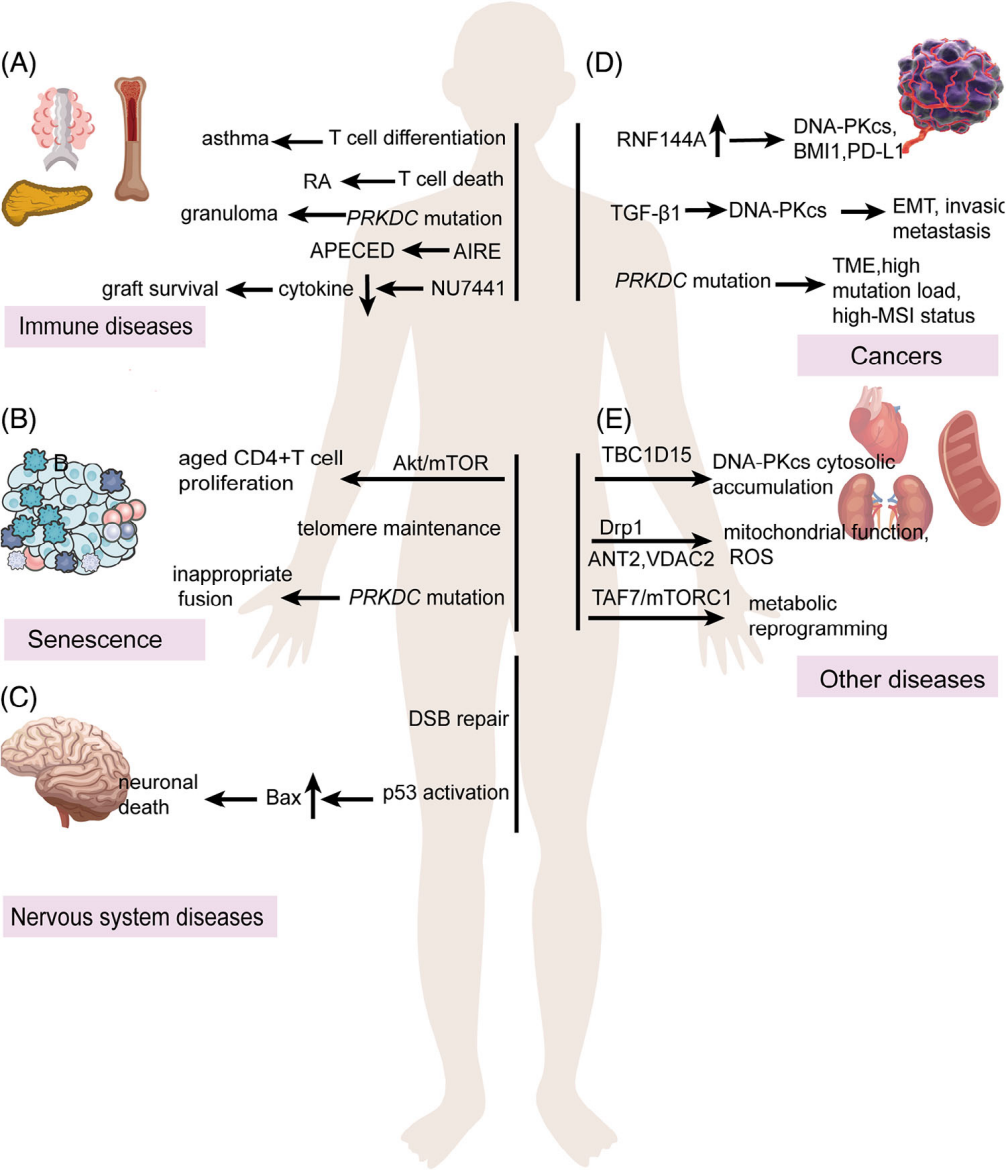
DNA‐PKcs in diseases and targeted therapies. (A) The function of DNA‐PKcs in immune‐related diseases. (B) The function of DNA‐PKcs in senescence. (C) The function of DNA‐PKcs in nervous system diseases. (D) The function of DNA‐PKcs in cancers. (E) The function of DNA‐PKcs in other diseases, such as cardiovascular disease and kidney diseases. RA, rheumatoid arthritis; APECED, autoimmune‐polyendocrinopathy‐candidiasis‐ectodermal dystrophy; PD‐L1, programmed cell death 1 ligand 1; EMT, epithelial–mesenchymal cell transformation; TME, tumor microenvironment; TBC1D15, TBC domain family member 15; ROS, reactive oxygen species; MSI, microsatellite instability. Created with BioRender.com (https://www.biorender.com).

In rheumatoid arthritis (RA), DNA‐PKcs transmits genotoxic stress into T cell that induces T cell death, premature immunosenescence, and the chronic proliferative turnover of immune system.^174^ DNA‐PK recruits AIRE to tissue‐restricted antigen (TRA) genes in chromatin. Lack of DNA‐PKcs abrogated the assembly and activity of AIRE to TRA genes, which is related with the autoimmune‐polyendocrinopathy‐candidiasis‐ectodermal dystrophy.^175^ Patients who were diagnosed with granuloma and autoimmunity with *PRKDC* mutations exhibited an incapability in DSB repair, V(D)J recombination, and a strong interferon signature.^60^ In asthma, the treatment of DNA‐PKcs inhibitor NU7441 blocks CD3 and CD28, induces T‐bet and Gata3 expression in CD4^+^ T cells, and stops Th1 and Th2 cells differentiation (Figure 5A).^166^ In organ transplantation, treatment of NU7441 significantly reduced extended graft survival and necrosis, reduced the production of IFN‐𝛾 IL2, IL4, IL6, IL10, and TNF‐α, and the infiltration of CD3^+^ lymphocytes, CD19^+^ B cells, and CD19^+^ CD138^+^ plasma cells.^171^ Evidently, DNA‐PKcs is emerging as an important regulator of the immune system and immune‐related diseases, spurring broad interest in its use as a therapeutic target. Targeting DNA‐PKcs in combination with other medications, such as tacrolimus and Janus kinase inhibitors, may also be useful for the treatment of organ transplant related rejection, rheumatologic disorders, and other immune‐related diseases.

### 3.2 DNA‐PKcs in senescence

Cellular senescence, first described in vitro in 1961, is prominently characterized by permanent proliferative arrest in response to endogenous and exogenous stresses, including DNA damage, telomere dysfunction, oncogene activation, and organelle stress, and has been implicated in processes such as tumor suppression, tissue repair, embryogenesis, and organ senescence. DNA‐PKcs also plays a key role in senescence. Loss of DNA‐PKcs shortens the life span of mice and causes an earlier onset of ageing‐related pathologies in mice.^176^ As for aged CD4^+^ T cells, Ku complex facilitated DNA‐PKcs recruits and ZAK phosphorylation, subsequently promoting CD4^+^ T cell expansion and activation via Akt and mTOR pathways.^68^ Knockdown of tankyrase 1 resulted in concomitant and rapid decrease of DNA‐PKcs, significantly elevated telomeres recombination, and accelerated cellular senescence. The same study showed that mutation in DNA‐PKcs was responsible for the telomere inappropriate fusion and dysfunction in SCID mice.^177^, ^178^ From the strand‐specific CO‐FISH technique, we can speculate that DNA‐PKcs might play a role at leading‐strand DNA synthesis, but the specific mechanisms need to further clarify.^179^ Another research further confirmed that DNA‐PKcs was required for Ku heterodimer functions on telomeres (Figure 5B).^178^, ^180^ But how and when phosphorylation of DNA‐PKcs promotes the DNA ends joining while preserving others remains an open and mysterious question, and whether or not partially inactivating DNA‐PKcs polymorphisms occur in humans has yet to be determined.

### 3.3 DNA‐PKcs in neurological disorders

Reduced DNA end‐joining is one of the characteristics of Alzheimer's disease (AD), along with reduced expressions of DNA‐PKcs and the Ku proteins.^181^, ^182^ Anatomically, Shackelford reported that NHEJ is decreased in cortical extracts and DNA‐PKcs expression was significantly reduced in the AD brain extracts.^183^ It is unknown whether HR changed in the AD. Postmortem studies suggest that p53 is abnormal in AD and the amino‐terminal site is phosphorylated by DNA‐PKcs. p53 promotes the proapoptotic protein Bax, ultimately leading to cell death. Meanwhile, Bax‐induced neuronal death also correlated with DNA‐PK‐mediated Ku70 phosphorylation.^184^, ^185^, ^186^, ^187^ On the other hand, DNA‐PK regulates the activities of RNA polymerase I/II in cell death and gene transcription.^188^ However, it is challenging to point out the exact roles of DNA‐PKcs and Ku80/Ku70 in AD. From a population‐based study, it is revealed that DNA‐PKcs was concentrated in temporal cortex and frontal cortex, and a good correlation was shown. Though p53 positive endothelial cells could be easily found within vessels, the correlation between DNA‐PKcs and p53 was not investigated.^189^ As we can see, the neuronal DNA damage response is identified at the earliest stages of AD, and DNA‐PKcs has a clarified relation to AD. In spinal muscular atrophy, low levels of survival motor neuron lead to Senataxin (SETX) deficiency and subsequent increased RNA–DNA hybrids (R‐loops) and decreased DNA‐PKcs, while overexpressed SETX could rescue neurodegeneration (Figure 5C).^190^ From these clinical trials, we can deduce that DNA‐PKcs plays an important role in nervous system diseases, and it can maintain the integrity of nerve cells to ensure normal physiological function. However, it is still necessary to further study the specific mechanism of DNA‐PKcs in nervous system diseases from the occurrence to the treatment, so as to develop more suitable therapeutic drugs.

### 3.4 Implications of DNA‐PKcs dysfunction in cancer development and progression

DNA‐PKcs frequently shows aberrant expression in cancers and plays critical roles in tumor metastasis and resistance to therapy and tumor immunotherapy.^191^, ^192^, ^193^, ^194^, ^195^ Tumors with high level *PRKDC* are enriched with IFN‐γ‐dominant and wound healing subtypes, and significantly correlate with CD8^+^ T cell and B cell signatures across different cancer types.^196^ Specifically, loss of RNF144A, the DNA damage‐induced ubiquitin E3 ligase, upregulates the expression of DNA‐PKcs, PD‐L1 and BMI1 that contribute to the bladder tumorigenesis.^197^ TGF‐β1 upregulates DNA‐PKcs expression and promotes cutaneous squamous cell carcinoma EMT program, invasion and metastasis.^198^ Solid tumors containing *PRKDC* mutations are abundant in TME, such as NK cells, CD8^+^ T cells, and chemokines.^199^ Recent study revealed that *PRKDC* mutation was corelated with high‐MSI status or high mutation load in cancers. Either *PRKDC* knockout or DNA‐PKcs inhibitor treatment enhanced the efficacy of ICI.^18^ Sueoka and coworkers^200^ found that adult T‐cell leukemia‐lymphoma cells have a high expression of DNA‐PKcs. NK314 induced DNA‐PKcs degradation and inhibited DSB repair. Paradoxically, DNA‐PKcs and ATM are useful for maintaining p53 phosphorylation in B and T cells and p53‐dependent apoptosis.^155^ DNA‐PKcs appears to act as a tumor suppressor in lymphoma, and DNA‐PKcs^−/−^ mice has high tendency to develop thymic lymphoma than wild‐type.^176^ DNA‐PKcs combines with PARP1 to suppress early development of T‐lineage lymphomas in mouse. Lacking of PARP1 and DNA‐PKcs in thymocytes leads to abnormal chromosome disjunction due to telomere fusions (TFs) and p53 inactivation.^201^ While EGFR amplification and TP53 mutation, the two prominent genetic alterations, show mutual exclusivity in glioblastoma multiforme (GBM), DNA‐PKcs was found to interact with p53 and inhibit p53 activity. EGFR suppressed wild type p53 activity through DNA‐PKcs binding with p53.^202^ Therefore, DNA‐PKcs exerted different biological functions at different cell developmental stages, and, in the case of whether p53 was mutated, DNA‐PKcs may exert opposite effects. DNA‐PKcs may exert different mechanisms in different oncogene contexts, and the mechanism of DNA‐PKcs action remains to be further explored under mutated or unmutated oncogenic context.

Interestingly, both our group and Adamson et al.^203^ showed that DNA‐PKcs can regulate the RNA alternative splicing.^204^ Aberrant androgen receptor (AR) signaling has proved to be the driver for prostate cancer (PC). However, AR splicing is demonstrated to be the one of the major mechanisms for therapeutic resistance or castration‐resistant PC. In Adamson et al.^203^ study, they discovered that DNA‐PKcs cooperates with RBMX to control global splicing and AR alternative splicing. Except for AR splicing, DNA‐PK also participated in metabolism in castration‐resistant PC (CRPC). Knudsen and coworkers^205^ group used metabolomic and proteomic approaches to identify the DNA‐PKcs protein interactome. They found that DNA‐PKcs interacts with ALDOA and PKM2 to regulates glycolysis. Combinational use of DNA‐PKcs inhibitor with glycolytic inhibitor 2‐deoxyglucose results in additive tumor‐suppressing effect.^205^

In human melanoma PDXs and cell lines, DNA‐PKcs inhibitor reduced the size of extrachromosomal DNAs and complex genomic rearrangements, thus preventing/delaying acquired MAPKi resistance early on combination treatment.^206^ A signature of BRCA1/2‐mutated tumors is the enrichment of stalled replication forks and replication‐associated genomic instability. In mammary tumors harboring BRCA2‐deficiency and acquired PARPi resistance, DNA‐PKcs inhibition prevents fork reversal and efficiently restores chemotherapy sensitivity.^207^ It is also reported that DNA‐PKcs inhibition acts synergistically with G‐quadruplexes inhibitor pyridostatin in eliminating BRCA1/2‐deficient cells and tumors.^208^ In glioma cells, dual targeting DNA‐PKcs and Flap endonuclease 1 synergistically stabilize replication fork to encounter replication stress.^209^ In clear cell renal cell carcinoma (ccRCC), the one‐carbon metabolic enzyme nicotinamide N‐methyltransferase induces DNA‐PKcs homocysteinylation, increased DNA repair, and enhanced ccRCC tumor growth.^210^ Overall, the definition of DNA‐PKcs seems to be more inclined to cancer promoting factor (Figure 5D).^8^

### 3.5 DNA‐PKcs in other diseases

Neurological disorders, immunodeficiencies, and cancers are the common features associated with DNA‐PKcs mutations or malfunctions, which are often linked to the nuclear DNA‐PKcs functions. Besides, DNA‐PKcs was also reported to mediate the doxorubicin (DOX)‐induced cardiotoxicity through interaction with TBC domain family member 15 (TBC1D15). TBC1D15 facilitated the cytosolic accumulation of DNA‐PKcs after DOX treatment.^211^ Moreover, cytoplasmic DNA‐PKcs plays an important role in the pathogenesis of cardiovascular diseases through its role in mitochondrial regulation.^212^ In DNA‐PKcs knockout Rat aortic smooth muscle cells (SMC) and VSMC‐specific model, angiotensin II provoked the accumulation of cytoplasmic DNA‐PKcs and subsequent interaction with dynamin‐related protein 1 (Drp1) though its TQ motif. DNA‐PKcs phosphorylates Drp1 at S616 site. Since Drp1 is critical in mitochondrial functions, DNA‐PKcs‐Drp1 interaction resulted in ROS production as well as mitochondrial fragmentation and dysfunction. In response to oxidative stress, DNA‐PKcs directly interacts with mitochondria proteins VDAC2 and ANT2 to promote optimal exchange of ADP and ATP across mitochondrial membranes to fuel the cell via oxidative phosphorylation and to maintain mitochondria membrane potential.^34^ DNA‐PKcs Thr2609 phosphorylation by ATM dissociates the complex of DNA‐PKcs/ANT2/VDAC2. In myogenesis, DNA‐PKcs interacts with the p300‐containing complex and activates transcription of Myogenin, affecting myogenic differentiation, myofiber composition and myogenesis upon injury.^213^ In both chronic kidney disease patients and unilateral ureteral obstruction and unilateral ischemia–reperfusion injury male mice model, DNA‐PKcs expression was found to be significantly increased.^214^ Targeting DNA‐PKcs can also correct metabolic reprogramming in injured epithelial cells and myofibroblasts through TAF7/mTORC1 signaling (Figure 5E).

## 4 DNA‐PKCS INHIBITORS AND THEIR THERAPEUTIC POTENTIAL

Based on extensive studies, DNA‐PKcs has become a hopeful therapeutic target, especially in combination with chemo‐ and radiotherapy in the treatment of tumors. A number of highly selective inhibitors such as AZD7648, NU7441, M3814, NK314, NU5455, and so on have been tested for cancers therapy.^27^, ^215^, ^216^ Specific inhibitors such as VX‐984, AZD7648 and M3814 have emerged and entered clinical trials. VX‐984 promotes the radiosensitivity of glioblastomas (GBM) and it can possibly permeate the blood‐brain barrier proving helpful in treatment of brain cancers.^217^ DNA‐PKcs inhibition by NU7441 also activates glioma stem cell differentiation and promotes glioblastoma sensitivity to radiation.^218^ VX‐984 combined with pegylated liposomal DOX (PLD) has been used in phase I trial.^219^ In Leiomyosarcoma, M3814 in combination with low‐dose DOX suppressed tumor growth both in vitro and in vivo.^220^ In ovarian cancer, the combination of PLD with M3814 increased the radiosensitivity of cells.^221^ M3814 in combinations with multiple therapeutic agents including radiation, etoposide, and DOX can gain excellent result rather than monotherapy. In non‐small cell lung cancer, M3814 enhances tumor chemosensitivity to paclitaxel/etoposide.^222^ Furthermore, preclinical studies show that NU7441 and M3814 treatment enhanced antitumorigenic effects of immunotherapy,^223^ and M3814 in combination with anti‐PD‐L1 antibody is testing in clinical trials. AZD7648, another specific inhibitor, increased the radiosensitivity and DOX‐induced DNA damage.^224^ Combination of Peptide receptor radionuclide therapy and AZD7648 significantly decreased viability of xenograft models of neuroendocrine tumor while showed hematologic and renal safety.^225^ The DNA repair inhibitor, AsiDNA has been used in solid tumors that combined with carboplatin and paxitaxel.^219^ AsiDNA can be designed to bind DNA‐PKcs and PARP‐1 because it can mimic DNA DSBs.^226^, ^227^ Together, these data show promising use of DNA‐PKcs targeted therapy to treat malignancies.

Low specific inhibitors targeting DNA‐PKcs are another choice. LY3023414, the PI3K/mTOR/DNA‐PK inhibitor, pleotropic modulator CC‐122 and CC‐115, the mTOR/DNA‐PK inhibitor, not only target DNA‐PKcs, but also suppress cancer relevance kinases. CC‐122 treatment showed encouraging results and longer survival.^228^, ^229^ Similar research pointed out that combination of Enzalutamide with CC‐115 made surprising results in CRPC patients, and all patients had at least a 50% decline of prostate‐specific antigen.^230^, ^231^ We enumerated the applications of DNA‐PKcs inhibitors in disease (Table 2) and its clinical application (Table 3). These studies provide promising indication that targeting DNA‐PKcs may be effective in eliciting antitumor effect. The next focus is how to improve the bioavailability of these inhibitors, extend the half‐life, and minimize the potential toxicity.

**TABLE 3:** DNA‐PKcs inhibitors in clinical trials.

NCT number	Study title	Patients condition	Interventions	Study status
NCT05002140	Trial of XRD‐0394, a Kinase Inhibitor, in Combination with Palliative Radiotherapy in Advanced Cancer Patients	Metastasis; locally advanced solid tumor; recurrent cancer	XRD‐0394 and palliative radiotherapy	Completed
NCT02316197	Clinical Phase I Study Investigating MSC2490484A, an Inhibitor of a DNA‐dependent Protein Kinase, in Advanced Solid Tumors or Chronic Lymphocytic Leukemia	Advanced solid tumors; chronic lymphocytic leukemia	MSC2490484A (M3814)	Completed

*Data sources*: https://clinicaltrials.gov.

PARP1, as the crucial member of the PAPRs family and one of the central players in DDR, has multifaceted roles in DNA repair, chromatin remodeling, gene transcription, ribosome biogenesis, and RNA biology. PARP inhibitors have been proved to be the most successful DDR inhibitor in clinic.^232^ What is more, increasing evidence are showing the overlapping roles of DNA‐PKcs and PARP1 in not only DDR, but also other processes including tumorigenesis, which provides the foundation of dual targeting these two master proteins. Without the Ku proteins, DNA‐PKcs and PARP1 cooperate to fulfill the alternative end joining.^233^ Upon DNA replication stress, DNA‐PKcs and PARP1 collaborate to recruit XRCC1 to maintain the genome stability at the stalled replication forks.^234^ In ETS gene fusions PC, TMPRSS2:ERG, interacts with DNA‐PKcs and PARP1,^235^ which are required for ERG‐mediated transcription. The TrpRS‐DNA‐PKcs‐PARP‐1 complex contributes to IFNγ and p53 signaling.^236^ In Mao and coworkers^237^ study, combinational targeting of DNA‐PKcs and PARP1 suppressed hepatocellular carcinoma (HCC) growth in both mice model and HCC patient‐derived‐xenograft model. DNA‐PKcs and PARP were also identified as mechanisms for the extrachromosomal DNA (ecDNA) amplification (also known as double minutes) in chromothripsis.^238^ Though the detailed mechanisms are unknow about how DNA‐PKcs and PARP make the ecDNA and whether the chromothripsis‐induced ecDNA is the common mechanism for all the chemotherapeutic drugs, noting that chromothriptically initiated rearrangements are often resulted from chemotherapeutic drugs, the combinational targeted therapy with both DNA‐PKcs and PARP inhibitors may represent an effective regimen to prevent cancer progression or chemo‐resistance.

## 5 CHALLENGES AND FUTURE DIRECTIONS

From its first discovery 30 years ago to the structure revelation, DNA‐PKcs has attracted widespread interest and attention for researchers. It was initially identified as component of the specificity protein 1 transcription complex and as a regulatory partner of transcriptionally poised RNA polymerase II.^246^ Until now it has been extensively investigated in DSB repair, especially in NHEJ. As a multifunctional protein, DNA‐PKcs participates in the activation and maturation of lymphocytes and are involved in tumors, autoimmune diseases, infectious diseases and aging processes (Figure 5).

DNA‐PKcs acts as a double‐edged sword. On one hand, DNA‐PKcs function is necessary for genomic integrity. DNA‐PKcs mediates Ig diversity to accommodate diverse antigenic stimulation, and DNA‐PKcs is involved in healthy body metabolism. As mentioned above, as a member of PIKKs family, the conserved kinase domain is located near the C‐terminus, where it is surrounded by regulatory domains such as the FAT, FATC, and PRD.^247^ The KU70/80 heterodimer and DNA‐PKcs assemble at the DSB ends to form the DNA‐PK holoenzyme.^246^ Researches have confirmed that S2056 and T3950 are two major autophosphorylation sites for DNA‐PKcs activity in DNA repair. DNA‐PKcs S2056 phosphorylation changes the conformation of DNA‐PKcs, promoting DNA‐PKcs disassociation from the DSB site, allowing DNA end ligation, while T3950 plays the opposite role, wherein its phosphorylation decease the activity of DNA‐PKcs kinase.^248^, ^249^ A recent study revealed that the nuclease Artemis could interact with DNA‐PKcs precisely. Mutation of DNA‐PKcs at L3062R impaired its interaction with Artemis and resulted in radiosensitive SCID, and mutations in two conserved regions of Artemis affected the interaction with DNA‐PKcs.^53^ There are so many sites of DNA‐PKcs need to clarify and other external mediators that interact with DNA‐PKcs should be verified.

On the other side, data from TCGA database indicate that *PRKDC* genetic alterations are related with poor prognosis in several cancer cohorts and DNA‐PKcs is defined as a protumorigenic protein kinase to repair DSBs that cause resistance to treatment. It promotes tumor growth, metastasis, invasion, and immune escape and regulates tumor metabolism.^250^, ^251^, ^252^, ^253^, ^254^ It is encouraging that many specific inhibitors such as NU7441, M3814, VX‐984, and AZD7648 can target DNA‐PKcs to reduce its kinase activity and control the development and progression of cancers from proliferation, differentiation, migration, and metabolism. Meanwhile, DNA‐PKcs in combination with IR, PLD, etoposide and so on have been tested in clinical trials. Promising results have been obtained. It is worth attention that safety assays, relevant toxicity assessments, and investigations of carcinogenesis risk prior to clinical application are missing for most inhibitors. Therefore, it is imperative to provide cancer patients with more selective, low toxicity, and higher efficiency combination treatments at a low cost.

Additionally, DNA‐PKcs can be applied as tool in CRISPR/Cas9‐based genome editing based on its dominant role in NHEJ. DNA‐PKcs inhibitor.^255^ AZD7648 significantly increased homology‐directed repair and decreased genomic insertions/deletions in nuclease‐based genome editing, implicating it as potent therapeutic approach in cell‐based therapies in the future. Using three fluorescent Cas9‐based reporters, Yaffe and coworkers^256^ found that DNA‐PKcs inhibition not only increases HR, but also mutagenic SSA, which is mediated by long‐range end resection factors DNA2 and Exo1. This should be considered and avoided when using genome editing.

Clearly, DNA‐PKcs has multifaceted functions, which are implicated in various physiological–pathological cellular events and human diseases. However, there are still many unresolved mysteries that need to clarify. Based on compelling evidence, it is worthy to investigate the DDR‐independent functions of DNA‐PKcs through in‐depth multi‐omics approaches. It is also interesting to explore DNA‐PKcs functions in different cellular compartments such as mitochondrial, ER/Golgi, and so on even phase separation. The significance of DNA‐PKcs in RNA metabolism is quite a mystery as we and other groups have proved that DNA‐PKcs is associated with RNA splicing, rRNA biogenesis, and so on. As for DNA‐PKcs classical role in DSB repair, how DNA‐PKcs coordinates with the resection machinery at the DSB sites remains an interesting question as well as the cross‐talks between DNA‐PKcs and ATM/ATR. The precise structural, physical, and chemical kinetics involved in conformation change of DNA‐PKcs during DDR is important too. Furthermore, how DNA‐PKcs impacts the higher‐order chromatin structure needs to be investigated. As a polypeptide, DNA‐PKcs contains 4128 amino acids, the sites function of PQR and ABCDE are clear, but other posttranslational modification sites should be explored to clarify their relationship and how they influence DNA‐PKcs functions. Above all, deciphering the temporal and spatial DNA‐PKcs interactome, and utilization of modern molecular biology technologies are the keys.

Regarding the aspects of DNA‐PKcs in immunity, some studies have pointed out the regulation of DNA‐PKcs on T and B cells, but the regulation on other types of immune cells is still lacking. As an important mediator during CSR and V(D)J, how and when DNA‐PKcs open hairpins? It is still unclear about how DNA‐PKcs cooperate with RAG1/RAG2/AID. It is also unknown if DNA‐PKcs is involved in SHM and in Ig pre‐mRNA maturation. Why IgG1 is unaffected in DNA‐PKcs^−/−^ mature B cells.

## 6 CONCLUSIONS

Clearly, DNA‐PKcs has multifaceted functions that are implicated in various physiological–pathological cellular events and human diseases. However, there are still many unresolved mysteries that need clarification. Although several DNA‐PKcs inhibitors have been developed, the DNA‐PKcs protein as a “giant” and a “Pandora” box for diseases targeted therapy needs further explorations. We need to identify more biomarkers for DNA‐PKcs inhibitors and explore, which chemotherapy drugs can be combined with DNA‐PKcs inhibitors. How and when to apply it to improve the effect of radiotherapy. Continuous exploration of DNA‐PKcs multiple roles and increasing the detection method in tumor, aging, autoimmune diseases, and infection diseases will greatly contribute to the discovery of new therapies and drugs.

## AUTHOR CONTRIBUTIONS

*Concept and design*: Teng Ma and Pingkun Zhou. *Data analysis and interpretation*: Jinghong Wu, Liwei Song, Mingjun Lu, Qing Gao, and Shaofa Xu. *Manuscript writing*: Jinghong Wu, Liwei Song, and Teng Ma. *Final approval of manuscript*: all authors.

## CONFLICT OF INTEREST STATEMENT

The authors declare no conflict of interest.

## ETHICS STATEMENT

Not applicable.

## Data Availability

All data and material are available in the main text.
